# Children and Young People's Priorities for Mental Health Research in Northern Ireland

**DOI:** 10.1111/hex.70295

**Published:** 2025-05-19

**Authors:** Siobhan O'Neill, Carol Rhonda Burns, Edel Ennis, Raymond Bond, Maurice Mulvenna, Elaine Murray, Thomas Wilson

**Affiliations:** ^1^ School of Psychology Ulster University Coleraine UK; ^2^ School of Geography and Environmental Sciences Ulster University Coleraine UK; ^3^ School of Computing Ulster University Belfast UK; ^4^ NI Centre for Stratified Medicine, Altnagelvin Hospital Campus Ulster University Derry UK; ^5^ Children's Learning Disability Therapeutic Service (Northern Health and Social Care Trust) Braid Valley Hospital Ballymena UK

**Keywords:** children, mental health, Northern Ireland, research priorities, young people

## Abstract

**Introduction:**

There are a number of factors contributing to the poor mental health of children and young people (CYP) specific to life in Northern Ireland (NI). Prevention and early intervention are of critical importance to the mental health and well‐being of CYP. Policy decisions and service provision in the health and education sectors must be informed by research so that we can understand the factors affecting the mental health of young people and develop effective policy responses. This study examines the perceptions of young people in NI regarding mental health research priorities.

**Methods:**

CYP who live in NI and are aged between 11 and 25 were invited to contribute to this priority setting exercise. A short anonymous online survey asked: ‘What do you think is the most important question that researchers should be trying to answer about the mental health and wellbeing of young people in NI? You may submit more than one question.’ Two‐hundred and seventy‐nine questions were submitted from 147 respondents. The priorities were then further discussed and expanded through focus groups with young people.

**Results:**

The study identified 12 research priorities. Using thematic analysis, these were grouped into four themes: (i) Ensuring that the voices of young people in NI are heard, (ii) Understanding and addressing the root causes, extent and impact of mental health challenges in young people, (iii) Creating accessible and effective youth mental health services in NI and (iv) Fostering a whole‐school approach to mental health and resilience.

**Conclusions:**

The research priorities of young people are discussed in relation to current governmental strategic policies and statistics. Suggestions are put forward regarding how these research priorities may be addressed.

## 1 Introduction

Mental health in childhood and adolescence is a predictor of educational attainment, physical health and improved psychological well‐being in adulthood [1, 2]. Poor mental health is a pressing and growing issue affecting the lives of many children and young people (CYP) in Northern Ireland (NI). One in eight young people experience emotional difficulties and one in ten have conduct problems, and 11.5% of young people (2–19 years old) may have a mental health disorder [3, 4].

Broad environmental factors and childhood adversities influence the development of mental illness in NI's young people. This includes poor childhood health, household dysfunction and living in deprived urban areas [4]. In addition, the Covid‐19 pandemic has disrupted young people's lives globally, to the detriment of their mental health [5]. In NI, the 30 years of civil conflict known as the ‘Troubles’ has also left a legacy of trauma among the adult population, which continues to influence the mental health of current younger generations. Widespread violence and exposure to conflict‐related events account for large portions of the disease burden of post‐traumatic stress disorder (PTSD) and complex post‐traumatic stress disorder (CPTSD) in adults in NI, which are among the highest in Western Europe [6]. Parental trauma and ill‐health may affect family functioning and parental attachment and result in childhood adversities [7, 8]. It may also lead to biological changes which increase the risk of poor mental health [9]. Deprivation is also a legacy of the conflict and has been shown to increase the risk of various mental health problems and suicidality in NI [10].

Patient and Public Involvement (PPI), wherein patients, caregivers and members of the public are actively engaged in the planning, design, delivery and evaluation of health research and services, is central to healthcare planning and design (McCabe et al. 2022; Totzeck 2024). PPI also supports patient engagement and patient‐centred care, both of which are associated with improved health outcomes [11, 12, 13]. Aligning with this, meaningful and effective co‐production and co‐design is a core principle of the Mental Health Strategy (2021–2031) [14].

In addition to being cognisant of this at a general population level, it is essential to ensure that the voices of young people are heard. Meaningful youth involvement is essential for addressing the key social, economic and environmental challenges affecting young people and their mental health [15]. Youth voices must be heard on issues that impact them [16]. As healthcare planning and design must be underpinned by evidence surrounding the factors affecting the lives and mental health of young people, establishing what young people identify as research priorities is a central part of this process.

Recognising these values and principles, the NI Executive's CYP's Strategy (2020–2030) outlines the need for frameworks to ensure young people are involved in the strategy's implementation. The NI Commissioner for CYP has also published guidance for public bodies on how to best facilitate young people's active and meaningful participation, which can act as a practical guide to encourage and enable public bodies to better involve service users in future [17].

In education settings, the recent ‘Children and Young People's Emotional Health and Wellbeing Education Framework’ [18] places a similar emphasis on creating opportunities for children's voices to be heard in ‘whole‐school’ approaches to well‐being and ensuring that young people are involved in decisions around their emotional well‐being.

While the need for involvement in policy and service decisions is described in theory, the extent to which this is implemented in practice is not so clear. The evidence for PPI activities with CYP is very sparse, and identification of the research needs of CYP represents an area of priority (McCabe et al. 2022; Totzeck et al. [12]). The picture appears similar within NI. The NI Commissioner for CYP's report, ‘Still Waiting’ [19], indicates that only 42% of young people agree that they are involved in decisions around their health.

The current paper reports the findings from a priority setting exercise, wherein young people were asked what they considered to be the most important question that researchers should be trying to answer about the mental health and well‐being of young people in NI. This exercise was undertaken within futureMINDS, which is a research network funded by the Medical Research Council. Findings will be mental health research priorities that have been developed in consultation with young people in NI. These main concerns have the potential to directly inform the development of research studies and funding calls.

## 2 Methods

### 2.1 Materials

Opinion gathering took two forms: an online consultation exercise and focus groups. For participant selection for the online consultation exercise, CYP who live in NI and are aged between 11 and 25 were invited to contribute to this priority setting exercise by responding to an online (Qualtrics) consultation with one open‐ended question. Participants were asked: ‘*What do you think is the most important question that researchers should be trying to answer about the mental health and wellbeing of young people in NI? You may submit more than one question.’* An option was also available for participants to select ‘*No thank you, I do not wish to submit a question’*. The online consultation exercise was followed up by two focus groups.

### 2.2 Participants

A total of 479 young people accessed the online consultation. Of those, 147 respondents submitted 279 valid comments/questions. It was possible for participants to submit more than one response. The remainder (*n* = 332) did not submit a question or make comments that were not in line with the consultation. Participation was anonymous, so no demographic details are available. Recruitment for the online consultation exercise took several forms. A link to the online consultation was shared with all students enrolled at Ulster University. In addition to this, several young people's organisations such as Youth Action NI, NI Youth Forum and other youth charities also disseminated the consultation through their social media channels and mailing lists. A link to the consultation was also shared across the futureMINDS social media presence.

The focus groups were convened with eight young people from the futureMINDS Research Network who agreed to take part in two focus groups (four in each) to discuss the initial findings from the consultation. As the online consultation had been anonymous, and the focus groups were only to interpret the findings from the online consultation, no demographic details were gathered.

### 2.3 Analysis

The exercise employed thematic analysis [20] to systematically organise and interpret the data. This yielded 12 research questions, which were grouped into 4 themes. Triangulation was employed through the involvement of multiple researchers and consideration of two data sources (online responses and clarification of the meaning of the questions through focus groups). This ensured credibility and robustness, resulting in a validated framework of research priorities reflecting consensus across perspectives.

In the initial phase of data analysis, one researcher grouped online consultation responses into broad topics. This step corresponded to the familiarisation and coding stages of thematic analysis, where patterns in the data were identified. The broad topics were then collaboratively classified into 12 research priority questions by two members of the research team. This step reflects the theme development phase of thematic analysis, where data is organised into overarching categories. Involving multiple researchers in coding and classification ensured researcher triangulation, reducing bias and incorporating diverse analytical perspectives to enhance the credibility of findings.

The 12 research priority questions were then refined through focus groups. Focus‐group discussions were conducted online and were recorded. The focus groups concentrated on gaining insights to refine the language, terminology and interpretations of the research priorities. These were employed as methodological triangulation wherein information from different methods (an online consultation exercise and focus groups) enhances the credibility, validity and robustness of findings. Finally, within theme refinement, four overarching themes of concern were identified within the 12 priority questions (all three members of the research team).

### 2.4 Ethical Approval

The consultation procedures were approved by the university's ethical committee. All information was managed in line with current legislation (General Data Protection Regulation [GDPR]). For both elements, informed consent was obtained. To encourage openness, anonymity was guaranteed for both elements, and no identifier information was gathered. They were made aware at the beginning that withdrawal of responses was not possible due to it being anonymous. Data storage is for 10 years.

### 2.5 Reflexivity

The research team, which consisted of two psychology academics and a government advisor on mental health services, was mindful of potential bias. To minimise the risk of bias due to their awareness of government priorities, the government advisor was not involved in interpreting the initial interpretation of the online data to identify the initial 12 research questions or in the data gathering stage of the focus group. Furthermore, the academic facilitator focused on creating a supportive environment to reduce power imbalances and encourage open expression. Multiple team members collaborated within each of the stages of reviewing the draft questions, integrating focus group feedback and conducting thematic analysis. This was to ensure participants' voices were accurately represented, minimising the influence of personal mental health experiences or biases.

## 3 Results

Table 1 presents the research priorities identified by the participants and agreed in the focus groups. These research priorities spanned four themes, with several research priorities being within three of these.

**Table 1:** Research priorities.

Theme 1: Ensuring that the voices of young people in NI are heard	Theme 2: Understanding and addressing the root causes of mental health challenges in young people	Theme 3: Creating accessible and effective Youth Mental Health Services in Northern Ireland	Theme 4: Fostering a whole‐school approach to mental health and resilience
Research priority 1: How can we ensure that the voices of young people in NI are heard?	Research priority 2: Identifying the unique and systemic factors affecting young people's mental health in Northern Ireland and developing targeted interventions to address these inequalities and support the overall well‐being of young people	Research priority 6: What are the factors that influence presentation to services or service access for young people in NI?	Research priority 10: How can mental health literacy, resilience and coping be integrated across the entire school curriculum at primary and post‐primary levels?
	Research priority 3: What can we do to make young people in NI feel less isolated, lonely or marginalised?	Research priority 7: How do we ensure mental health services are effective and meet the needs of young people?	Research priority 11: How can we reduce the impact of stress within the education setting for young people in NI?
	Research priority 4: How can we improve young people's engagement with and management of social media to maximise its positive impact and minimise its negative impact?	Research priority 8: What are the factors affecting perceived confidentiality in mental health and support services for young people in NI?	Research priority 12: What is the impact of a ‘whole‐school’ approach to mental health and well‐being?
	Research priority 5: Identification of the individual impact of mental health difficulties experienced by young people in NI	Research priority 9: What are the key components of person‐centred services?	

### 3.1 Theme 1: Ensuring That the Voices of Young People in NI Are Heard

*Research priority 1*:

*Identification and development of strategies and mechanisms to ensure that young people in Northern Ireland have meaningful opportunities to voice their perspectives.*

Young people emphasised the need for their voices to be central in health and education policy and service provision. They felt unheard on issues like service planning and development and highlighted the importance of understanding their role in shaping systems that impact them. Focus was placed on engaging marginalised youth and identifying effective ways to involve them in mental health policies and services.

Strategies and mechanisms can be developed to ensure that young people in NI have meaningful opportunities to voice their perspectives.

### 3.2 Theme 2: Understanding and Addressing the Root Causes, Extent and Impact of Mental Health Challenges in Young People

These research priorities are concerned with the broad factors influencing young people's mental health.

*Research priority 2*:

*Identifying the unique and systemic factors affecting young people's mental health in Northern Ireland and developing targeted interventions to address these inequalities and support the overall well‐being of young people.*

Young people acknowledged the significant impact of systemic factors such as socio‐economic inequalities, housing insecurity and limited access to education and resources on mental health. They highlighted unique challenges in NI, such as the legacy of conflict and societal divisions, and emphasised the need to understand how issues like social inequalities, loneliness and social media affect youth well‐being. They called for efforts to mitigate these effects through health and social care and addressing inequalities to better support those at risk.

*Research priority 3:*

*Identification of strategies to reduce isolation, loneliness and marginalisation among young people in Northern Ireland and foster a sense of connection and inclusion*.

Young people noted that while isolation is more common in minority and marginalised groups, it can affect anyone. They emphasised that meaningful social connections with friends and family are vital for psychological well‐being and identifying mental health issues. Understanding barriers to forming relationships and effective strategies to improve social connections could inform policies to reduce mental health risks. They felt that research should explore why young people feel isolated and how educational settings can foster safe environments for positive connections.

*Research priority 4:*

*Development of evidence‐based strategies to educate young people about engagement with social media in ways which enhance its positive effects while mitigating its negative impact on mental health and well‐being.*

Young people highlighted the benefits and challenges of social media for mental health, noting its role in fostering connections but also risks like misinformation, harmful content and promotion of disordered eating, self‐harm and suicide. They expressed a need for skills to manage social media but felt little is known about effective strategies to promote positive use. Research was deemed essential to guide young people, families and schools in navigating this complex issue.

*Research priority 5:*

*Identification of the individual impact of mental health difficulties experienced by young people in NI.*

Concerns were also raised about the lasting impact of poor adolescent mental health on individuals, including its effect on academic outcomes, future career prospects and overall life satisfaction. Participants emphasised that untreated mental health challenges during adolescence could hinder personal development, social relationships and long‐term well‐being.

### 3.3 Theme 3: Creating Accessible and Effective Youth Mental Health Services in NI

These research priorities are concerned with the process of accessing and experiencing support and evaluating aspects of those services.

*Research priority 6:*

*Identification of the factors that influence presentation to services or service access for young people in NI.*

In discussing young people's mental health, participants emphasised the need to understand factors affecting young people's access to mental health services and how services can better meet their needs. They identified stigma, difficulty finding suitable services and uncertainty about seeking help as key barriers. Understanding these factors was seen as essential for creating accessible services for young people's mental health.

*Research priority 7:*

*Identification of strategies for evaluating whether mental health services are effective and meet the needs of young people?*

Participants highlighted research about the quality and effectiveness of mental health services for CYP as a priority. Participants noted dissatisfaction among those who received care, feeling their needs were unmet, and emphasised the importance of improving assessments and treatments for young people's mental health. Current models were not considered to match the need.

*Research priority 8:*

*Identification of the factors affecting perceived confidentiality in mental health and support services for young people in NI.*

In discussing mental health services for young people, participants highlighted that concerns over confidentiality and privacy often deter young people from accessing mental health services. These concerns include data sharing in digital applications and the confidentiality of in‐person therapy. Young people emphasised the need to clearly communicate the limits of confidentiality, such as when information might be shared with parents. They called for research into effective ways of explaining confidentiality boundaries, how young people interpret them and their impact on trust and service effectiveness.

*Research priority 9:*

*Identification of the key components of person‐centred services.*

Young people felt services were not ‘person‐centred’, focusing narrowly on behaviours and symptoms instead of their identity, goals and circumstances. They emphasised the need for young people to be involved in designing, delivering and evaluating mental health services and suggested starting with young people defining ‘person‐centred’ care to guide service development.

### 3.4 Theme 4: Fostering a Whole‐School Approach to Mental Health and Resilience

These research priorities include a range of approaches to young people's mental health that span the curriculum, experiences within schools and those outside of school.

*Research priority 10:*

*Identification of how mental health literacy, resilience and coping can be integrated across the entire school curriculum at primary and post‐primary levels.*

Young people felt mental health education was inadequate and emphasised embedding mental health, resilience and coping skills into the school curriculum. They advocated for equipping children to understand and nurture their mental health and called for research into age‐appropriate approaches across educational stages. They also stressed the need to address the mental health education needs of diverse groups, including those with special educational needs (SEN) and from varying socio‐economic backgrounds.

*Research priority 11:*

*Identification of how we can reduce the impact of stress within the education setting for young people in NI.*

Young people noted that stress negatively affects mental health, well‐being and educational outcomes, with schools being a significant source. They called for research to identify modifiable stress factors within schools and the education system and to explore effective ways to reduce the impact of educational stress on young people.

*Research priority 12:*

*Identification of the impact of a ‘whole‐school’ approach to mental health and well‐being.*

Participants supported ‘whole‐school’ approaches to mental health, emphasising the integration of positive mental health throughout the school system, involving leaders, staff, pupils, parents and the wider community. They called for research to clarify the approach's characteristics and its impact on well‐being, mental health, academic outcomes and community connections. Adopting such approaches was seen as beneficial for reducing mental health risks, supporting staff and parents, improving behaviour and fostering broader community ties, with research needed to guide best practices in education.

## 4 Discussion

This project examined the mental health research priorities of young people in NI, which has been identified as an area of priority (McCabe et al. 2022; Totzeck et al. [12]). Twelve research priorities were established, which were categorised into four themes: (i) Ensuring that the voices of young people in NI are heard, (ii) Understanding and addressing the root causes, extent and impact of mental health challenges in young people, (iii) Creating accessible and effective youth mental health services in NI and (iv) Fostering a whole‐school approach to mental health and resilience. Although focused on mental health, these research priorities span the health and education sectors, both managed by separate NI Assembly departments with distinct budgets and priorities [21, 22]. Young people clearly see both as crucial to their mental health and well‐being.

### 4.1 Theme 1: Ensuring That the Voices of Young People in NI Are Heard

Research priority 1 focused on the need to understand how we can ensure that the voices of young people in NI are heard. Young people stressed the importance of having their voices central to health and education policies and service delivery. They expressed feeling overlooked in service planning and development and emphasised the need to recognise their role in shaping systems that affect them. They also highlighted the need to engage marginalised youth and explore effective strategies for involving them in mental health policy and service design. This finding aligns with the earlier NICCY report, *Still Waiting* (2018a), which revealed that only 42% of young people feel involved in decisions about their health. Despite PPI being a core principle of the *Mental Health Strategy (2021–2031)* [14] and evidence supporting its role in improving health outcomes through patient engagement and person‐centred care [11, 12, 13], young people report feeling that their voices are not adequately heard. This is concerning given the widely recognised importance of youth involvement [15, 16, 18] and the availability of practical guides designed to help public bodies effectively engage service users in research [17].

### 4.2 Theme 2: Understanding and Addressing the Root Causes of Mental Health Challenges in Young People

Research priority 2 highlights the need to identify the unique and systemic factors affecting young people's mental health in NI and develop targeted interventions to address inequalities and support well‐being. The Youth Wellbeing NI Survey [3] provided critical data on the prevalence of mental health problems and risk factors such as age, gender and deprivation, building on earlier findings like those in the *Still Waiting* report [19]. Young people emphasised aligning future research with existing data on lifestyle, loneliness, marginalisation and social media, stressing the need to ensure that findings are shared with the community and translated into tangible actions to address their mental health challenges.

While young people emphasised the importance of understanding and addressing the root causes of mental illness, they identified loneliness and marginalisation as key factors impacting their mental health. Research priority 3 focused on the need to identify what we can do to make young people in NI feel less isolated, lonely or marginalised. This aligns with existing research, which has shown that young people experience higher loneliness rates than other age groups [23]. This is significant given loneliness's strong link to physical and mental health [24]. This research priority emphasises the need to investigate and develop effective interventions specifically tailored to address loneliness and marginalisation among NI's most vulnerable young people.

While young people identified loneliness and marginalisation as key factors affecting their mental health, they also highlighted the dual impact of social media as a significant concern. Research priority 4 focused on understanding how we can improve young people's engagement with and management of social media to maximise its positive impact and minimise its negative impact. Echoing the Royal College of Psychiatrists [25], young people emphasised the need for further research on the benefits and risks of digital technology to develop strategies that promote safe, well‐being‐focused social media use. Related to understanding and addressing the root cause of mental health difficulties in young people in NI, research priority 5 focused on the need to study the individual impact of mental health difficulties experienced by young people in NI. Participants expressed concern about how poor adolescent mental health might affect an individual in the long term. For example, there is evidence that poor mental health in adolescence can influence education and employment outcomes and is associated with not being in employment or training. It is also linked with poorer physical health [26].

### 4.3 Theme 3: Creating Accessible and Effective Youth Mental Health Services in NI

Research priority 6 was where the young people noted a need to understand the factors that influence presentation to services or service access for young people in NI. The young people have highlighted stigma as a significant barrier to seeking mental health services. This aligns with existing research in the area. Stigma has been identified by young people and reports like the CYP's Strategic Partnership [27] as a key barrier to seeking mental health support. Other deterrents include safety concerns, confidentiality and practical issues such as time, transport and cost [19]. Currently, CAMHS does not record reasons for appointment non‐attendance, limiting insights into access barriers. While this data could help address challenges for those securing appointments, it overlooks barriers faced by those unable to access services at all, such as wait times, limited availability, referral difficulties, lack of awareness and systemic issues. To improve access to mental health services and improve timely support, comprehensive research is needed which addresses the aforementioned obstacles.

Research priority 7 emphasises the need to ensure mental health services effectively meet young people's needs. However, NI lacks a centralised database for CAMHS, limiting service evaluation and research. The *Still Waiting* report [19] highlights critical data gaps in demographics, needs, referrals, interventions and outcomes. This absence hampers efforts to assess intervention effectiveness, with 49% of young people reporting services as unhelpful. While the Mental Health Strategy [14] introduced a Regional Outcomes Framework, it excludes children's services. Establishing a robust data collection system is essential for evaluating CAMHS and improving outcomes for young people.

Research priority 8 emphasises the need to understand the factors affecting perceived confidentiality in mental health and support services for young people in NI. This aligns with the earlier‐mentioned discussions of stigma. Clear communication about confidentiality limits is crucial to maintaining trust, as breaches can damage relationships with professionals. Challenges arise when information is shared across families, schools and services with differing policies. Despite its importance, current health strategies do not address informing young people about privacy to reduce help‐seeking barriers. Research is needed to identify effective ways to navigate confidentiality, clarify when it may be broken and build trust with service users.

Research priority 9 emphasises the need to define key components of person‐centred services, addressing young people's concerns that current services often fail to meet their needs [19]. There is a need to further develop person‐centred services that focus on accessibility, delivery methods, decision‐making involvement, service locations and cultural competence to better address the diverse needs and identities of young people. Establishing ongoing, meaningful engagement with young people is crucial to building trust and improving service accessibility.

### 4.4 Theme 4: Fostering a Whole‐School Approach to Mental Health and Resilience

The Covid‐19 pandemic underscored schools' critical role in well‐being, as young people experienced significant declines in mental and physical health [5]. Schools provide multiple benefits, including academic learning, socialisation and safe spaces with trusted adults, as well as security, nutrition and routine.

Research priority 10 highlights the need to integrate mental health literacy, resilience and coping strategies into the primary and post‐primary curriculum. The *Children and Young People's Emotional Health and Wellbeing Education Framework* (2021) promotes a whole‐school approach to these concepts. Mental health literacy aids in recognising psychological distress [28], while resilience and coping skills help adaptation to challenges [29]. Effective integration empowers young people to support their own and others' mental health. However, school‐based interventions can sometimes unintentionally worsen symptoms in vulnerable students [30]. Research is needed to optimise delivery methods and mitigate potential harms.

To fully realise the potential of improved mental health education in schools, it is also important to examine the organisational and policy barriers or facilitators that may impact its implementation. For example, variations in funding, teacher training, curriculum priorities and available resources can significantly influence how effectively mental health education is integrated into schools. Additionally, policy‐level support, such as mandates for mental health education and collaboration between education and health sectors, plays a critical role in enabling schools to adopt and sustain such initiatives. Understanding these systemic factors is essential for ensuring that mental health education efforts are both scalable and equitable across diverse educational settings.

Research priority 11 focuses on reducing the impact of stress within the education setting for young people in NI. Participants highlighted concerns about academic pressures and their effect on mental health. The diathesis–stress model [31] explains how personal vulnerabilities and environmental stressors contribute to mental health issues. Research is needed to identify effective strategies to mitigate academic stress and support young people's well‐being.

Research priority 12 focused on examining the impact of a ‘whole‐school’ approach to mental health and well‐being. The *Framework for Emotional Wellbeing in Schools in NI* and the joint strategy from the Department of Health and Department of Education (2021) emphasise the importance of a whole‐school approach, integrating education and healthcare to support students' mental health. However, schools retain autonomy over the ways in which mental health education is delivered, and further research is needed to establish the impact of this policy.

## 5 Strengths and Limitations

Supporting the authenticity of the research process, this study has many of the features which McCabe et al. [32] outline as facilitators for youth engagement in mental health research (e.g., building relationships with community organisations and ensuring clear expectations). Through the anonymous online survey, the research also sought to have flexibility of engagement and a diversity of voices [32]. However, youth engagement in mental health research faces many challenges [32]. Whilst every effort was made to minimise the effects of these, their potential impact cannot be discounted. For example, we cannot be sure of sufficient diversity and the inclusion of marginalised groups.

An anonymous online consultation in NI may exclude marginalised groups, affecting the generalisability and reliability of findings. Young people without internet access, non‐native English speakers, neurodiverse individuals, those with disabilities or those in crisis may face barriers to participation. Similarly, younger adolescents, older youth or those disengaged from mental health discussions might be underrepresented, leading to a narrower range of perspectives. These exclusions risk biasing results toward those with fewer barriers. Future consultations should address these gaps through targeted outreach and improved accessibility.

## 6 Conclusion

The World Health Organisation (WHO) recognises adolescence as a critical life stage, emphasising the need for investment in adolescent health and the importance of youth partnerships in driving change [33]. This study identifies the research priorities of young people in NI, urging practitioners and policymakers to integrate these into public mental health strategies [34]. While this cannot be guaranteed, such approaches amplify the voices of marginalised groups. Notably, NI, as a post‐conflict society with high levels of deprivation, mental health challenges and transgenerational trauma [35], presents unique needs. Policymakers should consider how these priorities align with those in other international contexts. Examining young people's awareness of ongoing research and findings would be a valuable addition, as it would provide insight into the effectiveness of current communication strategies.

## Author Contributions

**Siobhan O'Neill:** conceptualisation, funding acquisition, writing – review and editing, methodology, project administration, supervision. **Carol Rhonda Burns:** writing – original draft, formal analysis, data curation, investigation. **Edel Ennis:** writing – original draft, supervision, formal analysis, writing – review and editing, methodology, funding acquisition. **Raymond Bond:** funding acquisition, writing – review and editing, project administration. **Maurice Mulvenna:** funding acquisition, writing – review and editing, project administration, supervision. **Elaine Murray:** funding acquisition, writing – review and editing, project administration, supervision. **Thomas Wilson:** data curation, formal analysis, writing – original draft, investigation.

## Ethics Statement

This study was approved by the ethics committee of the host institution. All procedures performed in studies involving human participants were in accordance with the ethical standards of the institutional research committee and with the 1964 Helsinki Declaration and later amendments or comparable ethical standards.

## Conflicts of Interest

The authors declare no conflicts of interest.

## Data Availability

The data that support the findings of this study are available from the corresponding author upon reasonable request.
